# Dosimetric comparison of intensity modulated and volumetric arc radiation therapy for gastric cancer

**DOI:** 10.3892/ol.2014.2363

**Published:** 2014-07-18

**Authors:** ZHIPING LI, JIANSHUANG ZENG, ZI WANG, HONG ZHU, YUQUAN WEI

**Affiliations:** 1 Department of Abdominal Oncology, Cancer Center of West China Hospital, Sichuan University, Chengdu, Sichuan 610041, P.R. China; 2 State Key Laboratory of Biotherapy and Cancer Center, West China Hospital, Sichuan University, Chengdu, Sichuan 610041, P.R. China

**Keywords:** gastric cancer, intensity-modulated radiotherapy, volumetric modulated arc therapy, dosimetric comparison

## Abstract

The aim of the present study was to compare radiotherapy treatment plans for gastric cancer using intensity-modulated radiotherapy (IMRT) and single/double-arc volumetric modulated arc therapy (SA/DA-VMAT) delivery techniques. A total of 29 postoperative gastric cancer patients were enrolled in this study and each patient was scheduled 5-field IMRT (5F-IMRT), 7-field IMRT (7F-IMRT), SA-VMAT and DA-VMAT techniques. Dose-volume histogram statistics, conformal index (CI), homogeneity index (HI) and monitor units (MUs) were analyzed to compare treatment plans. The DA-VMAT plans exceeded the other three methods in terms of planning tumor volume dose and organs at risk in the kidneys, but not in the liver. DA-VMAT exhibited a better mean CI (0.87±0.03) and HI (0.10±0.01) than the other techniques. In addition, for the kidneys the dose sparing (V13, V18 and mean kidney dose) was improved by DA-VMAT plans. Similar results were observed for MUs. However, 5F-IMRT showed a marginal advantage in V30 and mean dose in normal liver when compared with DA-VMAT. The results of this study suggest that DA-VMAT provides improved tumor coverage when compared with 5F-IMRT, 7F-IMRT and SA-VMAT; however, DA-VMAT exhibits no advantage in liver protection when compared with 5F-IMRT. Further studies are required to establish differences in treatment outcomes among the four technologies.

## Introduction

Gastric cancer is the fourth most common type of malignant tumor worldwide (1) and the annual number of novel cases is ~95 million. Each year ~70 million individuals succumb to gastric cancer, which makes it the second most common cause of cancer-related mortality worldwide (2). Since the SWOG/INT-0116 trial (3) in 2001, adjuvant chemoradiotherapy has become an established standard treatment for gastric cancer. In contrast to the INT-0116 trial, which included D0- or D1-resected gastric cancer patients, Kim *et al* (4) studied D2-resected participants using the same chemoradiotherapy regimens, and also demonstrated that concurrent chemotherapy increased survival and reduced recurrence.

However, compared with surgery alone, postoperative chemoradiotherapy significantly increased toxicity in patients. In the INT-0116 study, 57% of patients experienced grade 3 or 4 toxicity (3). Ringash *et al* (5) found that the application of three-dimensional conformal radiotherapy (3D-CRT) in patients with gastric cancer, which is different from the 2D radiotherapy used in the INT-0116 trial, decreased the incidence of grade 2 or higher toxicity to 25%. Similar studies have shown that conformal intensity-modulated radiation therapy (IMRT) achieves superior planning tumor volume (PTV) target coverage and improved normal tissue sparing (6–8). Furthermore, although the National Comprehensive Cancer Network Guidelines recommend either 3D-CRT or IMRT, it is now widely accepted in the medical profession that IMRT is superior to 3D-CRT in terms of tumor coverage, increased local tumor control probability and dose reduction to certain organs at risk (OARs).

Volumetric modulated arc therapy (VMAT), as a modified version of IMRT, employs the linear accelerators Elekta Synergy VMAT and Elekta Precise (Elekta Oncology Systems, Crawley, UK) to conduct dynamic modulation rotation radiotherapy. The advantages of VMAT when compared with IMRT, include a reduction in the number of monitor units (MUs), shorter delivery times and lower exposure of OARs. In practice, the VMAT optimization depends on the number of arcs and the gantry angle spacing between subsequent control points. At present, controversy exists as to whether a single arc VMAT can achieve dose distributions comparable to IMRT plans. Bertelsen *et al* (9) demonstrated that single arc is sufficient to achieve a plan quality similar to IMRT, however, Guckenberger *et al* (10) have reported that it is dependent on the complexity of the target volume.

VMAT is considered to be equivalent or superior to IMRT for certain malignancies, including head and neck, prostate, lung, cervical and pancreatic cancer (11), however, a lack of comprehensive comparison between IMRT to VMAT exists with regards to gastric cancer treatment. Therefore, the present study aimed to elucidate the dosimetric quality of single-arc (SA)/double-arc (DA)-VMAT for gastric cancer, compared with 5-field IMRT (5F-IMRT) and 7-field IMRT (7F-IMRT).

## Patients and methods

### Patient samples

A total of 29 patients with nonmetastatic gastric or gastroesophageal (GE) junction cancer who received radiotherapy treatment at the West China Hospital (Chengdu, China) between February 2012 and August 2012 were included in the study. All patients were confirmed by pathology and disease was limited to the stomach or GE junction and regional lymph nodes. Patients were staged according to the American Joint Committee on Cancer staging system (7th edition) (12). Each patient was rescheduled retrospectively via inverse planning 5F-IMRT, 7F-IMRT, SA-VMAT and DA-VMAT techniques using Pinnacle treatment planning systems (TPS; Philips Medical Systems, Fitchburg, WI, USA). Patient characteristics are summarized in Table I. Patients provided written informed consent.

### Immobilization, simulation and target delineation

All patients were immobilized in a supine position, with arms crossed above the head using a thermoplastic shell. Intravenous contrast-enhanced computed tomography (CT)-simulation was performed at 3 mm intervals of abdomen using Gemini GXL positron emission tomography/CT (Philips Medical Systems). Respiratory control and abdominal compression were not used. Following simulation, the CT images were transferred to the Pinnacle3 version 9.2 radiation treatment planning system (Philips Medical Systems). The clinical target volume (CTV) included tumor bed and perigastric lymph nodes, following the recommendations outlined in the INT-0116 trial (3). Paracardial, splentichilum, paraaortic, celiac, paraesophageal, hepatoduodenal and pancreaticoduodenal cancer celiac, as well as paracardial, paraaortic, celiac, paraesophageal, hepatic portal, pancreaticoduodenal and splenic hilum lymph nodes were included if deemed as high risk, based on the pathologically involved regional lymph nodes and the primary tumor location. The CTV to PTV expansion was typically 5–10 mm to account for daily setup error and organ motion. Normal structures, including the spinal cord, liver, colon, duodenum, small intestine and kidneys were also contoured. All the contours were drawn by the same physician. Each patient had one 5F-IMRT, one 7F-IMRT, one SA-VMAT and one DA-VMAT plan created by the same radiation therapist. The same dose constraints were used for creation of 5F-IMRT, 7F-IMRT, SA-VMAT and DA-VMAT plans (Table II).

All generated plans for each patient consisted of 50.4 Gy to be delivered to PTV in 28 fractions. The objective of planning was to deliver the prescribed dose to ≥95% of the PTV with a dose range that did not exceed −10 and +15% of the prescribed dose. All plans were generated for the Elekta Beam Modulator (Elekta Oncology Systems).

### Treatment planning and optimization; 5F-IMRT and 7F-IMRT

The IMRT optimization was performed using the direct machine parameter optimization algorithm in the treatment planning system (Pinnacle3; Philips Radiation Oncology Systems). IMRT uses five and seven coplanar beams; five beam beam irradiation, angles of 25, 60, 95, 180 and 315°; or seven bean irradiation, angles of 0, 51, 102, 153, 204, 255 and 306°. In the plan generation, the maximum iterations in the plan optimization were 80. There were no limitations with regard to the MUs per segment. Plans were generated for the Elekta Beam Modulator with 6-MV.

### SA-VMAT

The single arc VMAT planning was performed using the SmartArc planning algorithm in Pinnacle3 version 9.2 (Philips Radiation Oncology Systems). The single arc VMAT was planned with a beam delivery time ≤240 sec, and with an arc from 181–180° (a control point every 4°). The accelerator used automatic dose rate selection, which ensured that the maximal possible dose rate was selected for each individual segment of the arc. The initial step was performed using the SmartArc algorithm to obtain the optimal modulated fluency. In the second step, the segments were optimized based on the small target areas receiving insufficient irradiation dose, using the same algorithm. Plans were generated with 6-MV.

### DA-VMAT

The plans were optimized in the same planning system as mentioned previously. The double arc VMAT was planned with a beam delivery time of ≤120 sec x2, and with a gantry rotation of 181-180-181° (a control point every 4°). Plans were generated with 6-MV and all the objective parameters and algorithm used were the same as that for the single arc VMAT. All the plans were repeatedly optimized until the objectives were met.

### Evaluation of the dose-volumetric histogram (DVH)-based parameters

For the PTV, D_98_, D_95_, D_50_ and D_2_%, where D is the accepting dose and n is the percentage of the PTV, were selected to comply with the International Commission on Radiation Units and Measurements Report No. 83 (13). The conformal index (CI) and homogeneity index (HI) for PTV were calculated. The CI was defined as follows: CI = cover factor (the percentage of the PTV volume receiving 50.4 Gy) × spill factor (the volume of the PTV receiving the 50.4 Gy relative to the total prescription dose-volume). The HI was defined as follows: HI = the minimum dose in 5% of the PTV (D_5_)/minimum dose in 95% of the PTV (D_95_). The following dosimetric parameters were retrospectively analyzed: Volumes of kidney receiving a dose of ≥13 and 18 Gy (V13 and V18); volumes of liver receiving a dose of ≥30 Gy; D_2_ of the spinal cord; volumes of small intestine and colon receiving a dose of ≥50 Gy (V50); the mean dose to OARs and remaining volume at risk; the maximum dose to 1, 5 and 10 cm^3^ of the pancreas and duodenum; and the volume of pancreas and duodenum receiving 5, 10, 15, 20, 25, 30, 35, 40, 45, and 50 Gy.

### Statistical analysis

The data were analyzed using SPSS software, version 16.0 (SPSS, Inc., Chicago, IL, USA) and all data are presented as the mean ± standard deviation. The Wilcoxon’s signed rank test was performed and P<0.05 was considered to indicate a statistically significant difference.

## Results

### PTV coverage

**Figure 1:**
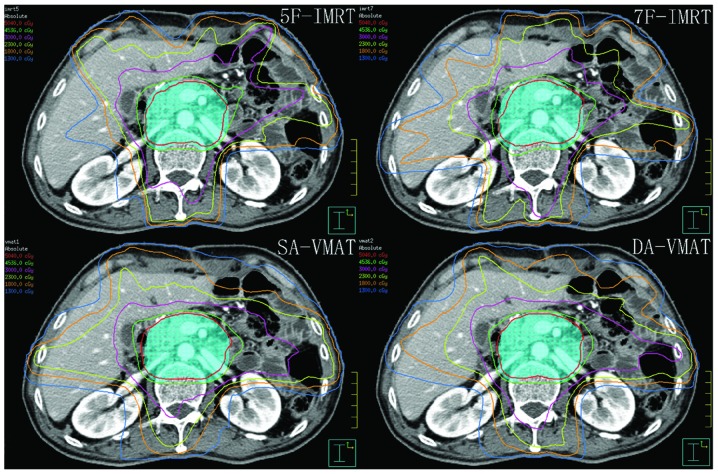
Dose distribution in a typical transverse slice. Planning tumor volume is presented as light-blue color wash. The red, green, pink, yellow, orange and indigo lines represent isodose curves of 50.4, 45.36, 30, 23, 18 and 13 Gy, respectively.

The evaluation of the DVH-based parameters of the PTV is shown in Table III. The D_98_ and D_95_ of the PTV were similar among the 5F-IMRT, 7F-IMRT, SA-VMAT and DA-VMAT plans, respectively, and no significant differences were identified (P>0.05). For the PTV coverage, the mean CI of the DA-VMAT plans (0.87±0.03) was significantly higher than that of 5-IMRT (0.86±0.02), 7-IMRT (0.86±0.02) and SA-VMAT (0.83±0.03), respectively (P<0.05). Additionally, the mean HI of the DA-VMAT plans (0.10±0.01) was found to be significantly improved when compared with those in 5-IMRT (0.13±0.17), 7-IMRT (0.10±0.02) and SA-VMAT (0.12±0.02), respectively (P<0.05). DA-VMAT plans also exhibited a lower D2 (54.21±49.92) when compared with the 5-IMRT (54.52±43.27), 7-IMRT (54.54±57.63) and SA-VMAT (55.33±109.69) plans (P<0.05). A typical dose distribution in the transverse section is shown in Fig. 1.

### OARs

DA-VMAT significantly decreased the mean dose (14.44±157.59 Gy), V13 (0.36±0.04 Gy) and V18 (0.26±0.03 Gy) of the left kidney. Similarly, a lower mean dose (11.23±188.43 Gy), V13 (0.27±0.06 Gy) and V18 (0.17±0.05 Gy) were observed in the contralateral kidney with DA-VMAT. The mean doses to the normal liver for each method were 21.90±138.97 Gy (DA-VMAT), 23.42±194.66 Gy (SA-VMAT), 21.91±147.73 Gy (7F-IMRT) and 19.82±196.08 Gy (5F-IMRT), with the mean dose to the normal liver with 5F-IMRT found to be the lowest. Furthermore, the V30 Gy (%) with SA-VMAT (0.22±0.05) was higher than that with 5F-IMRT (0.19± 0.03) (P<0.05), 7F-IMRT (0.19±0.03)(P<0.05) and DA-VMAT (0.19±0.03)(P<0.05). The results are shown in Table IV.

For the other OARs (Table V), no significant differences in dose were identified among the four methods, with the exception of the marginal edge in D2 for the duodenum with DA-VMAT and D1, D5 and D10 cm^3^ for the pancreas with DA-VMAT. The maximum dose to the spinal cord (D2) was equal for all four methods.

The VMAT plans were applied with fewer MUs (346.10±44.94 MUs for SA-VMAT and 437.66±62.69 MUs for DA-VMAT) than the efficient 5F-IMRT plans (456.41±89.50 MUs), while the 7F-IMRT required more MUs (578.55±97.98 MUs).

## Discussion

As mentioned previously, adjuvant chemoradiotherapy for resectable gastric adenocarcinoma has become the standard treatment for D0 and D2 gastrectomy. However, due to the combination of radiotherapy and chemotherapy, treatment-associated toxicities are enhanced, which often leads to relinquishment of treatment among patients. A number of studies on dosimetric comparison of 3D-CRT and IMRT have shown that IMRT exhibits improved OAR sparing. Few studies have investigated the application of VMAT in treating postoperative gastric cancer patients (14).

It is known that the complexity of the target volume and the number of VMAT arcs are major determinants of whether VMAT is advantageous when compared with IMRT (11). In contrast to the studies on head and neck cancer mentioned previously (9,15), certain studies on cervical cancer (16) and benign intra-cranial tumors (17) have demonstrated that SA-VMAT is superior or equivalent to IMRT. However, in contrast to these studies, less complexity was identified in target volume for gastric cancer with one dose level than that for head and neck cancer with two or three dose levels (15,18). In addition, the OARs in gastric cancer radiotherapy were found to be more radiosensitive than that in cervix uteri radiotherapy (18,19). As expected, the data in the present study indicated that the treatment planning for gastric cancer DA-VMAT plans achieved superior dose coverage for PTV (CI and HI were improved; P<0.05). Regarding HI, SA-VMAT exhibited an advantage when compared with 5F-IMRT, but not 7F-IMRT. For the CI, the SA-VMAT exhibited no advantage when compared with IMRT.

It is known that the kidney is a radiosensitive organ and that damage to the kidneys is an inevitable side effect of pelvic or abdominal radiotherapy. Previous studies (20,21) have suggested that total doses of 18–23 Gy and 28 Gy in 0.5–1.25 Gy/fractions may be associated with a 5 and 50% risk of injury in five years, respectively. Jansen *et al* (22) conducted a prospective study analyzing kidney function in 44 gastric cancer patients following abdominal irradiation and observed an 11 and 52% decrease in left renal function after six months and 18 months, respectively. The V20 (left kidney) and mean left kidney dose were identified as parameters associated with decreased kidney function. Therefore, in the present study V13 and V18 Gy were selected as indicators. The doses to the kidneys were significantly decreased in DA-VMAT plans; however, the V13 Gy, V18 Gy and D_mean_ in the left kidney were generally higher than those of the right kidney. One reason for this may be that the majority of the left kidney is located in the superior section of the target volume. In order to optimize dose distribution in the tumor bed, which is anterior to the left kidney, it is difficult for TPS to reduce the irradiation dose to the left kidney. By contrast, the right kidney is located in the lower section which is the paraaortic lymph node region. Since it is much smaller and more regular than the upper section, it is easier to complete dose computation.

For patients in China, radiation-induced liver disease (RILD) must be considered. As a parallel organ, the radiation injury to the liver is found to positively correlate with the volume and dosage of radiation to the normal hepatic tissues. Emami *et al* (23) reported that TD5/5 (the tolerance dose leading to a 5% complication rate at five years) for one-third, two-thirds and the whole liver at one dose of 8–2 Gy/day were 50, 35 and 30 Gy, respectively. However, these data were predominantly obtained from clinical practice in North America and may not apply to the situation in China. Based on a national seroepidemiological survey, the carrier rate of HBsAg in China among 1- to 59-year-olds is 7.18% and among the medically examined individuals in Chengdu, the HBsAg positive rate is 6.1% (24). At present, there is no constraint on the standard dose for those vulnerable patients.

The incidence of RILD is significantly associated with mean dose to normal liver (MDTNL) which may be a predictor of RILD. In a study that investigated the dose-volume tolerance for RILD using the Lyman-Kutcher-Burman normal tissue complication probability model, it was found that no cases of RILD were identified when the mean liver dose was <31 Gy (25). Each 1 Gy increase in MDTNL exhibited a 4% increase in the incidence of RILD. Furthermore, at an MDTNL of 43 Gy the incidence was as high as 50%. Liang *et al* (26) demonstrated that when the MDTNL was 23 and 31 Gy, the RILD occurrence rate was 6 and 69%, respectively. In addition, a MDTNL of 23 Gy may be used as a predictor of RILD. Furthermore, the risk of hepatitis B virus (HBV) radiotherapy reactivation has been identified, which must be considered. Previous study has revealed that radiotherapy is a significant risk factor to RILD in patients with postgastrectomy adenocarcinoma carrying HBV (27). For those patients, a reduction in volume and dosage of radiation to the normal hepatic tissues, as well as frequent monitoring of liver function and routine detection of the HBV-DNA copy numbers, are required. If necessary, regular antiviral treatment should be provided. In the present study, only the MDTNL of SA-VMAT plans exceeded 23 Gy and 5F-IMRT plans provided improved sparing of the liver with a marginal advantage when compared with SA-VMAT.

In addition, the dosimetric parameters of the duodenum and pancreas were compared among the four technologies. Anatomically, the duodenum is the first section of the small intestine. However, in the practice of radiotherapy, they are different with regard to dose and volume limit. As the duodenum adjoins the stomach, the majority of it is located within the target volume. Additionally, as the duodenum is fixed by the ligament of Treitz, the motion of the duodenum is more limited than that of the rest of the small bowel. In a dose escalation trial of pancreatic cancer, Singh *et al* (28) revealed that the volume of duodenum receiving a dose of >80% of the prescribed dose was greater than the remaining small bowel; however, individual variations were significant. Therefore, reducing the dose received by the duodenum is an important issue. Severe gastrointestinal (GI) toxicity appears to be the main dose-limiting factor in abdominal radiotherapy and it may be one of the reasons why the IMRT is superior to 3D-CRT in terms of OARs sparing. However, in practice, there is no difference in acute GI toxicity grade 2 between IMRT and 3D-CRT. In a previous study, the acute grade 2 or greater GI toxicity was found to be 61.5 and 61.2% for 3D-CRT and IMRT, respectively (7). According to Liu *et al* (14), the acute toxicity was 56 and 54% for the IMRT and 3D-CRT groups, respectively. At present, studies investigating dose constraints of the duodenum are rare. Huang *et al* (29) have suggested that the V25 Gy of the duodenum is the best predictor for GI toxicity in pancreatic cancer patients with concurrent gemcitabine-erlotinib and radiotherapy. The 12-month GI toxicity rates were found to be 8 and 48% for V25 Gy ≤45% and V25 Gy≥ 45%, respectively (P=0.03). Excluding the erlotinib group, the V35 Gy was the best predictor and the 12-month GI toxicity rates were 0 and 41% for V35 Gy ≤20% and V35 Gy ≥20%, respectively (P=0.04). Although chemotherapeutics are different in the treatment of gastric cancer and pancreatic cancer, the indicators remain useful. In conclusion, in the present study, all four technologies reached the standard for the indicator of V25 Gy ≤45%, however, for V35 Gy≤0%, all technologies failed. This analysis is only a preliminary step and, thus, further study is required to improve the sparing of the duodenum and identify dosimetric predictors for GI toxicity.

The manner in which the pancreas may be protected during abdominal radiotherapy is another issue which remains unclear. Similar to the duodenum, the motion of the pancreas is limited and the majority of it is within the target volume. Radiation induced damage of the pancreas predominantly decreases the endocrine and exocrine functions of the pancreas (30,31). In the present study, DA-VMAT was found to be marginally more effective than the other three technologies in D1, 5 and 10 cm^3^.

Regarding the issue of how to improve the sparing of OARs, other options are available. Hu *et al* (32) reported that the dose for postoperative gastric cancer patients may be increased to 54 Gy without increasing the toxicity to critical organs, by using a combination of breath-holding techniques and online image-guided IMRT. However, the problem of controlling gastric emptying remains; patients do not always follow doctor’s advice to ensure the GI tract is empty during the course of radiotherapy.

In conclusion, although VMAT has been demonstrated to exhibit advantages in the treatment of other kinds of malignancies, the dosimetric advantage of VMAT in this study was not always evident when compared with IMRT. In addition, it is unclear whether IMRT should be replaced by VMAT. Considering the lower MUs, shorter delivery times and reduced low-dose exposure of OARs, the use of VMAT in postoperative radiotherapy remains suitable for gastric carcinoma; however, the clinical implications and outcome require further study.

## Figures and Tables

**Table I:** Patient characteristics.

Parameters	Patients, n (%)
Gender	
Male	24 (83)
Female	5 (17)
Grade	
Well-differentiated	0 (0)
Moderately differentiated	3 (10)
Poorly differentiated	26 (90)
Clinical T Classification	
T1	1 (3)
T2	3 (10)
T3	15 (52)
T4	10 (35)
Clinical N Classification	
N0	1 (3)
Nl	5 (17)
N2	11 (38)
N3	12 (42)
Location	
GE junction	0 (0)
Cardia/proximal one-third	10 (34.5)
Body/middle one-third	10 (34.5)
Antrum/distal one-third	9 (31)
Surgery	
Total gastrectomy	13 (45)
Subtotal gastrectomy	13 (45)
Proximal gastrectomy	3 (10)
Ivor-Lewis esophagectomy and proximal gastrectomy	0 (0)
Total esophagogastrectomy	0 (0)

Median age of patients at diagnosis was 53.31 years (range, 24–73 years) and the median number of lymph nodes dissected was 23 (range, 10–51). Total number of positive lymph nodes found were 6 (range, 0–16) and the percentage of positive lymph nodes was found to be 27% (range, 0–59%). GE, gastroesophageal.

**Table II:** OARs dose constraints.

OARs	Prescribed dose limit
Spinal Cord	D_max_<40 Gy
Liver	V30<30%
Kidney	V13<50%
	V18<33%
Small intestine	D_max_<50 Gy
	V50<10%
	V45<15%
Duodenum	D_max_<50 Gy
	V50<10%
	V45<15%

V_n_, percentage of volume receving at least x Gy; OARs, organs at risk.

**Table III:** Comparisons of the dose-volume histogram-based parameters of the planning tumor volume.

	Radiotherapy				P-value					
		
Prameters	5F-IMRT	7F-IMRT	SA-VMAT	DA-VMAT	5F-IMRT vs. 7F-IMRT	5F-IMRT vs. SA-VMAT	5F-IMRT vs. DA-VMAT	7F-IMRT vs. SA-VMAT	7F-IMRT vs. DA-VMAT	SA-VMAT vs. DA-VMAT
D_98_, Gy	49.10±0.57	49.12±0.59	49.06±0.51	49.11±0.46	0.680	0.363	0.624	0.180	0.280	0.524
D_95_, Gy	50.35±0.44	50.34±0.41	50.41±0.38	54.21±0.50	0.870	0.104	0.446	0.070	0.480	0.380
D_50_, Gy	51.10±8.81	52.60±0.50	53.43±0.54	52.81±0.43	0.219	<0.001	0.944	<0.001	0.380	<0.001
D_2_, Gy	54.52±0.43	54.54±0.57	55.33±1.10	54.21±0.50	0.834	<0.001	0.001	<0.001	<0.001	<0.001
CI	0.86±0.02	0.86±0.02	0.83±0.03	0.87±0.03	0.994	<0.001	0.012	<0.001	0.010	<0.001
HI	0.13±0.17	0.10±0.02	0.12±0.02	0.10±0.01	0.715	0.003	0.013	<0.001	0.030	<0.001

n=29. CI, conformal index; HI, homogeneity index; 5F-IMRT, 5-field intensity-modulated radiotherapy; 7F-IMRT, 7-field intensity-modulated radiotherapy; SA-VMAT, single-arc volumetric modulated arc therapy; DA-VMAT, double-arc volumetric modulated arc therapy.

**Table IV:** Comparisons of the dose-volume hisotgram-based parameters of the kidneys and liver in present study.

					P-value					
					
OARs	5F-IMRT	7F-IMRT	SA-VMAT	DA-VMAT	5F-IMRT vs. 7F-IMRT	5F-IMRT vs. SA-VMAT	5F-IMRT vs. DA-VMAT	7F-IMRT vs. SA-VMAT	7F-IMRT vs. DA-VMAT	SA-VMAT vs. DA-VMAT
Left kidney										
V13	0.38±0.05	0.39±0.04	0.41±0.04	0.36±0.04	0.539	0.039	0.010	0.075	0.001	<0.001
V18	0.27±0.04	0.28±0.04	0.29±0.05	0.26±0.03	0.586	0.038	0.211	0.068	0.104	0.001
Mean dose, Gy	15.42±1.93	14.96±1.91	15.68±1.98	14.44±1.58	0.159	0.680	0.003	0.153	0.080	0.010
Right kidney										
V13	0.36±0.06	0.34±0.05	0.33±0.05	0.27±0.06	0.053	0.016	<0.001	0.479	<0.001	<0.001
V18	0.19±0.06	0.23±0.04	0.22±0.06	0.17±0.05	0.005	0.046	0.179	0.549	<0.001	0.002
Mean dose, Gy	12.83±2.15	12.83±2.03	12.62±2.10	11.23±1.88	0.680	0.529	0.001	0.780	0.001	0.008
Liver										
V30	0.19±0.03	0.19±0.03	0.22±0.05	0.19±0.03	0.592	0.013	0.895	0.038	0.762	0.018
Mean dose, Gy	19.82±1.96	21.92±1.48	23.42±1.95	21.90±1.39	<0.001	<0.001	<0.001	0.002	0.721	0.001

5F-IMRT, 5-field intensity-modulated radiotherapy; 7F-IMRT, 7-field intensity-modulated radiotherapy; SA-VMAT, single-arc volumetric modulated arc therapy; DA-VMAT, double-arc volumetric modulated arc therapy.

**Table V:** Comparisons of the dose-volume historgram-based parameters of the other organs at risk and MUs in present study.

					P-value					
					
OARs	5F-IMRT	7F-IMRT	SA-VMAT	DA-VMAT	5F-IMRT vs. 7F-IMRT	5F-IMRT vs. SA-VMAT	5F-IMRT vs. DA-VMAT	7F-IMRT vs. SA-VMAT	7F-IMRT vs. DA-VMAT	SA-VMAT vs. DA-VMAT
Small intestine										
D_2_, Gy	48.22±9.46	48.67±9.43	49.55±9.53	48.58±9.38	0.499	0.044	0.686	0.137	0.846	0.107
V50%	0.04±0.03	0.04±0.03	0.05±0.03	0.04±0.03	0.721	0.237	0.750	0.312	0.889	0.327
Mean dose, Gy	17.19±6.26	16.50±5.76	18.01±6.34	16.90±5.92	0.658	0.613	0.858	0.301	0.762	0.388
Colon										
D_2_, Gy	51.38±2.87	51.54±2.94	52.51± 2.47	51.46±2.93	0.703	0.018	0.715	0.040	0.932	0.042
V50%	0.09±0.07	0.09±0.07	0.11±0.08	0.09±0.07	0.926	0.316	0.895	0.423	0.883	0.347
Mean dose, Gy	22.39±5.07	21.47±4.42	23.50±5.66	22.57±5.19	0.539	0.397	0.950	0.159	0.451	0.451
Spinal cord										
D_2_, Gy	38.98±2.41	38.90±2.29	39.08± 4.44	38.50± 3.18	0.950	0.432	0.686	0.410	0.762	0.347
Pancreas										
D1cm^3^, Gy	54.52±0.70	54.51±1.03	55.38± 1.16	54.13± 0.57	0.834	0.002	0.004	0.005	0.003	<0.001
D5cm^3^, Gy	53.92±0.47	54.02±0.60	54.84±0.99	53.73±0.54	0.646	<0.001	0.059	<0.001	0.006	<0.001
D10cm^3^, Gy	53.53±0.48	53.70±0.56	54.50±0.94	53.47±0.54	0.312	<0.001	0.280	<0.001	0.024	<0.001
V5%	0.99±0.01	0.99±0.02	0.97±0.17	1.00±0.01	0.601	0.975	0.992	0.601	0.580	0.983
V10%	0.98±0.05	0.98±0.06	0.98±0.06	0.98±0.06	0.885	0.680	0.687	0.809	0.830	0.970
V15%	0.97±0.08	0.97±0.08	0.98±0.08	0.97±0.09	0.639	0.664	0.841	0.986	0.782	0.795
V20%	0.97±0.09	0.97±0.09	0.97±0.09	0.97±0.09	0.696	0.935	0.872	0.626	0.845	0.788
V25%	0.96±0.10	0.94±0.13	0.96±0.09	0.96±0.10	0.738	0.961	1.000	0.816	0.713	0.852
V30%	0.94±0.10	0.94±0.10	0.95±0.10	0.95±0.10	0.797	0.845	0.743	0.685	0.477	0.814
V35%	0.92±0.11	0.92±0.11	0.93±0.11	0.93±0.11	0.907	0.785	0.618	0.767	0.613	0.767
V40%	0.89±0.12	0.89±0.12	0.90±0.12	0.90±0.12	0.907	0.709	0.756	0.658	0.785	0.919
V45%	0.85±0.13	0.85±0.13	0.86±0.13	0.86±0.13	0.969	0.504	0.803	0.608	0.876	0.810
V50%	0.76±0.16	0.77±0.15	0.78±0.15	0.78±0.15	0.870	0.570	0.774	0.703	0.797	0.810
Mean dose, Gy	48.02±7.12	48.15±6.82	49.93±4.52	49.27±4.42	0.969	0.138	0.602	0.146	0.750	0.216
Duodenum										
D1cm^3^, Gy	53.11±1.59	53.15± 1.36	53.93±1.77	53.02± 1.41	0.762	0.036	0.534	0.036	0.786	0.017
D5cm^3^, Gy	50.84±4.20	51.03±3.30	51.79±4.36	50.83±4.41	0.703	0.041	0.994	0.020	0.738	0.039
D10cm^3^, Gy	46.97±9.57	46.87±9.19	48.05±9.49	46.78±9.97	0.715	0.174	0.994	0.107	0.663	0.225
V5%	0.88±0.16	0.88±0.16	0.89±0.15	0.89±0.16	0.890	0.942	0.961	0.821	0.885	0.859
V10%	0.83±0.19	0.83±0.19	0.84±0.18	0.83±0.19	0.912	0.869	0.950	0.832	0.912	0.826
V15%	0.81±0.21	0.81±0.20	0.82±0.20	0.81±0.20	0.895	0.761	0.839	0.679	0.851	0.778
V20%	0.76±0.22	0.76±0.21	0.78±0.22	0.77±0.23	0.926	0.554	0.697	0.427	0.709	0.634
V25%	0.72±0.23	0.73±0.22	0.75±0.23	0.73±0.24	0.864	0.437	0.726	0.470	0.870	0.619
V30%	0.67±0.24	0.68±0.23	0.70±0.24	0.67±0.24	0.858	0.524	0.994	0.646	0.846	0.565
V35%	0.61±0.24	0.61±0.24	0.65±0.25	0.62±0.25	0.932	0.570	0.981	0.581	0.969	0.504
V40%	0.55±0.24	0.55±0.24	0.58±0.25	0.56±0.25	0.981	0.669	0.957	0.658	0.907	0.646
V45%	0.48±0.24	0.48±0.24	0.50±0.24	0.49±0.24	0.969	0.680	0.932	0.680	0.889	0.797
V50%	0.37±0.23	0.38±0.23	0.40±0.25	0.38±0.23	0.944	0.560	0.726	0.635	0.944	0.750
Mean dose, Gy	36.12±10.39	36.18±10.15	37.53±10.66	36.41±10.56	0.994	0.451	0.907	0.423	0.883	0.539
MUs	4.56±0.90	5.79±0.98	3.46±0.45	4.37±0.63	<0.001	<0.001	0.721	<0.001	<0.001	<0.001
RVR, Gy	20.69±1.51	21.04±2.11	21.18±1.90	20.51±1.57	0.339	0.414	0.613	0.944	0.148	0.222

MUs, monitor units; RVR, remaining volume at risk. 5F-IMRT, 5-field intensity-modulated radiotherapy; 7F-IMRT, 7-field intensity-modulated radiotherapy; SA-VMAT, single-arc volumetric modulated arc therapy; DA-VMAT, double-arc volumetric modulated arc therapy.
